# Visual contrast sensitivity in patients with impairment of functional independence after stroke

**DOI:** 10.1186/1471-2377-12-90

**Published:** 2012-09-12

**Authors:** Natanael Antonio dos Santos, Suellen Marinho Andrade

**Affiliations:** 1 Laboratório de Percepção, Neurociências e Comportamento, Departamento de Psicologia, Universidade Federal da Paraíba, João Pessoa, Brazil

**Keywords:** Contrast sensitivity, Stroke, Functional independence, Spatial frequency

## Abstract

**Background:**

Stroke has been considered a serious public health problem in many countries, accounting for complex disorders involving perception, such as visual, cognitive and functional deficits. The impact of stroke on the visual perception of individuals with impairments in functional independence was investigated.

**Methods:**

We measured changes in functional independence and visual function in 40 patients with stroke (M = 52.3, SD = 0.65) and 10 controls (M = 52.5, SD = 0.66). The patients were divided into four subgroups following the Barthel Index (Group A: 20–35, serious dependence; Group B: 40–55, moderate dependence; Group C: 60–95, mild dependence; and Group D: 100 points, independence). Visual function was evaluated using the Contrast Sensitivity Function (CSF). The contrast threshold was measured using a temporal, two-alternative, forced-choice psychophysical method.

**Results:**

The results show significant differences in CSF between healthy volunteers and patients with stroke (F (1.56) = 151.2, p < 0.001) for all frequencies (F (2.56) = 125.96, p < 0.001). The results also show that patients with low functional independence had lower contrast sensitivity than those with greater functional independence (F (3.56) = 344.82, p < 0.001).

**Conclusions:**

An association exists between CSF and a worsening in the functional potential for performing daily living activities. Our results suggest that the CSF can be used as a diagnostic tool to analyze visual function associated with deficits in functional independence after stroke. These findings should be considered across the continuum of care for these patients.

## Background

Stroke can be defined as a focal neurological deficit of presumed vascular origin that starts suddenly and lasts for at least 24 hours, or until death if the patient dies within 24 hours after the onset of symptoms [1]. Stroke is the main cause of death and disability in many countries, mainly among medium and low-income individuals, accounting for more than 85% of worldwide mortality [2].

Stroke represents the main cause of functional impairment in the superior and inferior limbs of the injured patients and negatively affects their performance in the activities of daily living. In 30 to 60% of cases, the upper limbs continue to be disturbed in function for up to 6 months after the stroke. After this time, only 5 to 20% of patients recover full mobility [3].

In addition to losses in functional capacity, patients may present with deficits in perception, demonstrating psychomotor, cognitive and visual impairment. The main problems with visual function related to vascular lesions are hemianopsy, amaurosis, hemispatial neglect and visual agnosia. Such dysfunctions may reciprocally interact with patients’ functional capacity [4].

Visual cognitive neuroscience, through non-invasive imaging techniques such as Contrast Sensitivity Function (CSF), can objectively assess attributes of visual perception that are reduced after vascular lesion. The CSF is a classic instrument that is used as a clinical and theoretical indicator of vision [5] and has been frequently used to assess and diagnose deficits resulting from changes in the retina [6], visual pathways [7] and even disorders that are not directly related to impairments in the visual system, such as Parkinson’s disease [8], multiple sclerosis [9,10] and schizophrenia [11]. Currently, the CSF has been rarely studied in stroke patients [12], although it has importance as an objective tool because it is easy to apply and can be used as a diagnostic method for visual perceptual disorders and monitoring of the evolutionary process of stroke patients [13].

A lack of self-correction of visual control in order to detect different levels of contrast may cause processing changes in the integration of visual information with other systems, resulting in a decrease in the adaptability of an individual to the basic requirements of daily life [14]. In a study using the Visual Function Quality of Life Questionnaire, Zhao et al. [15] found a strong association between quality of life and visual function in patients who underwent cataract surgery, demonstrating the impact of loss of visual ability for patients. Analyzing 175 patients with brain damage, Lew et al. [16] found that visual impairment contributed to reduced gains in total (t = −2.25) and motor (t = −2.50) FIM (Functional Independence Measure) scores (p < 0.05) and that visual impairment may be detrimental to functional recovery during the rehabilitation process.

Thus, the objective of this study was to analyze the thresholds of contrast sensitivity in patients after stroke having different levels of functional independence, using low, medium and high spatial frequencies, and to compare their performance with a group of healthy individuals.

## Methods

A retrospective, ex post facto study was performed using a repeated measures design. All participants were subjected to the same randomly presented spatial frequencies. The design was formed by two independent variables (spatial frequencies and group) and one dependent variable (threshold or contrast sensitivity).

### Participants

Fifty volunteers participated in this study. These volunteers were selected for their accessibility and age (between 40 and 65 years old). Forty of the volunteer patients had suffered a stroke (the Experimental Group, EG, (M = 51.02, SD = 0.65)), and 10 were healthy volunteers (the Control Group, CG, (M = 52.5, SD = 0.66)). The patients were divided into four subgroups, each with 10 participants, according to their level of functional independence, which was determined based on the Barthel Index: Experimental Group A (EGA), 20–35 points, serious dependence; Experimental Group B (EGB), 40–55 points, moderate dependence; Experimental Group C (EGC), 60–95 points, mild dependence; and Experimental Group D (EGD), 100 points, independence [17]. There were 5 women and 5 men in each group (CG, EGA, EGB, EGC, and EGD). Table 1 presents the demographic and clinical data of the participants.

**Table 1:** Personal data and neuropsychological assessment (Mean±SD) arranged by group

**Group**	**CG**	**EGA**	**EGB**	**EGC**	**EGD**	**Post hoc p**
Age	51.7±2.8	51.3±2.2	50.2±1.8	50.6±2.5	52.0±2.6	ns
Education (years)	6.4±2.1	5.7±1.4	6.1±1.9	5.1±2.6	5.3±1.5	ns
Frontal Assessment	17.3±1.3	16.5±0.7	16.1±0.3	16.7±0.4	16.4±0.6	ns
Mini Mental State Examination	29.2±0.2	20.7±1.1	21.8±1.6	24.3±1.2	24.6±1.9	ns

CG: Control Group; EGA: Experimental Group A; EGB: Experimental Group B; EGC: Experimental Group C; EGD: Experimental Group D; ns: not significant.

The inclusion criteria adopted in the study included diagnosis of non-recurring unilateral ischemic stroke, acute stage (occurring at least one month after the vascular event), having injuries to the middle cerebral artery and normal or corrected visual acuity. Data were obtained from medical records and Functional Resonance Magnetic Imaging (fMRI). Participants in the Control Group were healthy individuals who were accompanying the patients or working in the institution where the experiment was performed. The pathology diagnosis was performed based on ICD-10 (International Statistical Classification of Diseases and Related Health Problems).

Exclusion criteria were hemorrhagic stroke, recurring, extensive cerebral lesion, incapability of completing the interview and assessment due to serious aphasia, psychiatric dysfunctions, ocular diseases, unconsciousness or use of drugs that modulate activity of the central nervous system, serious functional independence, with total dependence corresponding to a score lower than 15 on the Barthel Index.

All participants underwent prior ophthalmologic assessment, which included anamnesis and ophthalmological examination. The examination included: determination of visual acuity, external examination, anterior segment biomicroscopy, tonometry, gonioscopy, fundus examination and determination of biometric eye data, such as corneal curvature (keratometry), axial diameter (biometry), corneal thickness (pachymetry) and corneal diameter.

Only participants with visual acuity or corrected acuity, as analyzed by the Snellen chart, and who were not suffering from eye diseases were admitted to the study. This exclusion reduces the possibilty of creating bias in the comparison, because variables such as degree of stromal hydration, presence of cataracts, glaucoma, or even changes in intraocular pressure can have a very significant effect on visual function and may affect interpretation of the data. There were no changes in the visual field of the participants. The presence of hemineglect was evaluated using the Letter Cancellation Test (p>0.05).

Table 2 presents the average ocular biometric data; based on t-tests, there were no significant differences between healthy subjects and patients.

**Table 2:** Differences between the means of groups (Control and Experimental) in relation to axial diameter, corneal diameter, central corneal thickness and keratometry

**Categories**	**Group**	
	**t**	**p**
Axial Diameter	0.43	0.54
Corneal Diameter	0.21	0.84
Central Corneal Thickness	0.17	1.20
Keratometry	0.49	0.93

Comparison between means was performed using Student's t test and the respective p values.

Functional dependence was assessed based on the Barthel Index. This index measures the level of assistance demanded by an individual in 10 items of Activities of Daily Living (ADL), which involve mobility and personal care. The levels of measurement range from complete independence to the need for assistance. Each item of performance is assessed on an ordinal scale, and a specific number of points is marked for each level or classification. Different measures were established for each item, based on clinical judgment and other implicit criteria. Example scores include: 0 (dependent), 5 (need for help or supervision), 10 (partially dependent) and 15 (independent). The latter score is considered only for chair and bed transference or ambulation. In total, there are 100 possible points that define the level of dependence of a person (0 – 15 = total dependence; 20 – 35 = serious dependence; 40–55 = moderate dependence; 60 – 95 = mild dependence; and 100 = independent). This scale, which was developed for assessing the level of dependence in elderly patients or those who have serious sequels, for instance stroke, has been used since 1965, and its use is recommended by the World Health Organization (WHO) because it is easy to apply and can be adapted to different cultures [18].

For the Control Group, a Cumulative Illness Research Scale (CIRS) was applied to guarantee the participation of healthy individuals in this group. This scale investigates the presence of 14 disease sets (cardiac, vascular, hematological, respiratory, ocular, upper and lower gastrointestinal tract, hepatic and pancreatic, renal, genitourinary, musculoskeletal and integumental, neurologic, endocrine-metabolic, breast and psychiatric), taking into consideration situations in which each set of diseases is absent, mild, moderate, severe or extremely severe, with scores ranging from 0 to 4, respectively [19].

The participants were informed about the study protocol and the objective of the experiment. Subjects signed informed consent forms according to Resolution n° 196/96 of the Brazilian National Health Council (Health Ministry, Brazil), which determines guidelines for research involving human beings in compliance with the Declaration of Helsinki. The local Ethics Committee approved this research (Protocol # 249/09).

### Equipment and stimuli

The stimuli were set to appear in the center of a 19-inch video monitor (LG) CRT (Cathode Ray Tube) with high resolution (1024 x 768) and a 70-Hz frame rate. Input was controlled by a microcomputer through a video board with VGA and DVI connectors. The voltage luminance of the monitor was expanded from 8 to 14 bits using BITS++ (Cambridge Research Systems, Rochester, Kent, England, 2002), allowing the use of visual stimuli with lower contrast gradations. LightScan software, equipped with OptiCAL Photometry (Cambridge Research Systems, Rochester, Kent, England, 2002), was used to measure screen luminance and gamma correct the monitor using 48 index values ranging from 0 to 255 (gamma = 1.8) as a sample. The lowest and highest luminance values of the screen were 0.20 cd/m^2^ and 80.0 cd/m^2^ (mean luminance = 40.1 cd/m^2^). The room was 2.5 x 2.0 m in size and was illuminated by a fluorescent 20 W bulb (Philips). The walls of the room were gray, which allowed for better control of the room lightning conditions during the experiment. A C++ computer program, developed by the responsible lab, was used to run the experiment (generating the stimuli, controlling stimuli presentation and registering contrast thresholds).

**Figure 1:**
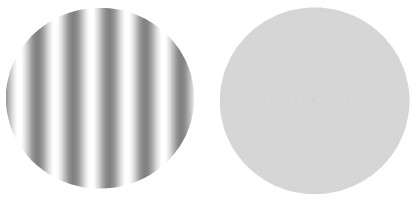
**Example of a pair of stimuli.** On the left, a sine-wave grating with the spatial frequency of 0.6 cpd, and on the right, a neutral stimulus. The stimuli were originally calibrated for viewing at a distance of 150 cm.

Achromatic and vertical static sine-wave grating stimuli with spatial frequencies of 0.6, 2.5 and 10.0 cycles per degree (cpd) of visual angle were used in this work (Figure 1).

All of the stimuli had a diameter of approximately 7.2 degrees of visual angle and were designed to be presented in the middle of the monitor at a distance of 150 cm from the observer.

### Procedure

Estimates were obtained using the psychophysical method of forced choice between two temporal alternatives [20]. This method is based on the probability of consecutive correct choices being made by participants; that is, in 100 presentations of a choice between two stimuli, the spatial frequency (test stimulus) is perceived by the participant in **7**9% of the presentations. The procedure for measuring the threshold for each frequency was successive presentation of pairs of stimuli, from which the participant had to choose which had the spatial frequency of interest. The other stimulus was always a homogeneous pattern of mean luminance 40.1 cd/m2. The criterion adopted to vary the contrast of each tested spatial frequency was three consecutive correct choices for decreasing by unity and an incorrect choice for increasing by the same unity (20%) [21].

An experimental session began with a brief beep followed by the presentation of a 2 s stimulus (test or neutral) with an inter-stimulus interval of 1 s and presentation of the second stimulus for 2 s, followed by the volunteers’ response. The interval between stimuli pairs and trials was 3 s. A different beep gave feedback to the volunteer for each correct choice. The order of presentation of the stimuli was random.

The participants received the following instructions: “pairs of stimuli, one with clear-dark stripes and another totally gray, will be presented. You must always choose the stimulus that contains the stripes, pressing the button on the left (button number 1) of the mouse when the stimulus with stripes is presented first, and the button on the right (button number 2) when it is presented in the second place (after the gray stimulus)”. Each session began with the test stimulus contrast at supra-threshold level, and the experiments began only when the investigator was convinced that the participant understood the directions and responded as instructed.

The experimental session varied in duration (with an average time ranging from 10 to 15 minutes) depending on the correct and incorrect choices made by the volunteer, until a total of six reversals (or six contrast values, three peaks and three valleys) were obtained. Each one of the points (or frequencies) of the contrast sensitivity curve was estimated at least twice (two experimental sessions), on different days, for each of the participants. All of the estimates were measured with binocular vision and natural pupil.

Descriptive statistics (Mean and SD) were used to analyze the clinical and demographic groups. A two-tailed Student t-test for paired samples was used to verify differences between the ocular conditions of control subjects and affected individuals. We used Analysis of Variance (ANOVA) to determine whether there were significant differences between the groups. Simple differences between treatments were adjusted using a post hoc Tukey HSD test, and the level of significance was p < 0.05.

## Results

**Figure 2:**
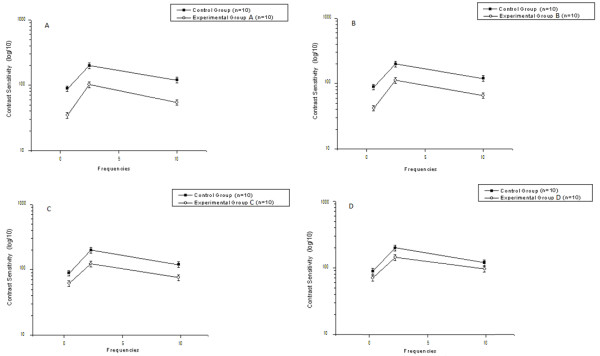
**Contrast sensitivity to sine-wave gratings.** Contrast Sensitivity Curves for Spatial Frequencies of Patients with Functional Independence, according to the Barthel Index, from 20–35 (2**A**), 40–55 (2**B**), 60–95 (2**C**) and 100 points (2**D**). The letter “n” represents the number of curves measured for each sub-group. The vertical lines show the standard error of the mean for each frequency (0.6, 2.5 and 10.0 cpd).

After each session, the program produced a result sheet with the experimental results and the contrast values obtained by the reversals. Contrast values for each frequency were grouped into spreadsheets by condition, either Experimental Group (EG) or Control Group (CG), and the mean value was used as an estimate of the contrast sensitivity to each tested spatial frequency.

(Figure 2) displays the contrast sensitivity curve for spatial frequencies for each Experimental Group (A, B, C, and D) compared to the Control Group. The contrast threshold can be defined as 1/CSF, i.e., the inverse of the contrast sensitivity curve. Thus, high threshold values indicate low sensitivity and vice versa. The vertical bars in the figure indicate the standard error of the mean with 99% confidence intervals, corrected for sample size using Student’s t-test.

The contrast sensitivity curves present similar profiles. All of the groups obtained a maximum sensitivity of 2.5 cpd spatial frequency, with reductions in the extremes of the curves. The participants in the Control Group were more sensitive than the participants in Experimental Groups A, B, C and D, with spatial frequencies of 2.9; 2.7; 2.4 and 2.1, respectively. These values were calculated as the arithmetic mean of the differences for detecting the stimulus contrast, such as the sinusoidal sine-wave grating, for the three tested frequencies. It was observed that an increase in functional independence, represented by a higher score on the Barthel Index, corresponded to a higher sensitivity in the detection of spatial frequencies when compared to all experimental groups.

The ANOVA showed significant differences between the condition (Experimental or Control Group, F (1.56) = 151.23, p < 0.001), between frequencies (F (2.56) = 125.96, p < 0.001), between functional independence (F (3.56) = 344.82, p < 0.001), and significant interactions of frequency versus condition (F (2.56) = 35.67, p < 0.001), frequency versus functional independence (F (3.56) = 52.46, p < 0.001), and frequency versus condition versus functional independence (F (3.56) = 20.37, p < 0.001). The post hoc Tukey HSD showed that patient groups A, B, C and D presented higher contrast sensitivity for sine-wave grating than the Control Group for all tested frequencies (p < 0.001).

## Discussion

The data showed that contrast sensitivity for sine-wave grating was better in the Control Group than in the Experimental Group, suggesting that healthy individuals have mean contrast thresholds that are higher than those of patients who have suffered a stroke. Changes in the CFS after stroke were also verified in a study by Bullens et al. [22], in which contrast sensitivity was analyzed in 16 patients with unilateral ischemic lesions involving posterior visual space. The authors observed that 62% of the patients presented a loss of contrast sensitivity for the orientation of horizontal or vertical stimuli. The authors also observed that visual perception was distorted in a qualitatively different way that depended on the anterior-posterior location of the lesion.

The results of this study showed that the maximum contrast sensitivity at photopic levels of luminance occurred at the frequency of 2.5 cpd for sine-wave grating in both groups, with a reduction of sensitivity at the extremes of the curves. These results, which were obtained with a luminance of 40.1 cd/m^2^, when compared to the data found in the literature [23], show that using photopic luminance moves the maximum sensitivity zone to the right of the CSF. The classic definition for a CFS function is an upside-down “U”-shaped curve, as found in similar studies that used Cartesian visual stimuli [24].

The results suggested differences between the degrees of functional independence and an interaction between frequency and functional independence. Overall, these data indicate an association between CSF and a worsening in the functional potential for performing activities of daily living. The results match data obtained by Wolter and Preda [25], who found that patients may present deficits in contrast sensitivity after stroke that can affect their independence for visual perception or adaptation to the environment. These patients generally complain that there is not enough light or that there is too much light in the environment, and this contrast sensitivity can cause problems with glow and the contour detection of objects. This phenomenon is frequently related to functional and neurological compromise, such as difficulty in performing activities of daily living, reading and mobility tasks.

Neuropsychological and psychophysical studies show that stimuli, such as sine-wave gratings, are processed by the primary visual cortex (V1) [26]. Thus, the results obtained show a relationship between the degree of functional incapacity resulting from stroke and the compromise of the visual area involved in the processing of such stimuli.

Studies on the attention and detection of spatial frequencies suggest that cortical mechanisms are involved in the selection of specialized information for high and low spatial frequencies. However, such processes appear to be related to specific neural operations and are dependent on the affected area and the type of task presented [27,28]. Thus, electrophysiological and neuroimaging studies involving demands from different locations and types of tasks could contribute to the understanding of the mechanisms of information processing involving injury to the right and left hemispheres.

Unfortunately, our study did not have sufficient power to stratify and analyze patients according to the mechanisms through which the injuries occurred. Further studies comparing inpatient rehabilitation populations could address this issue.

The CSF is related to age, and aging can cause the onset of degenerative eye diseases. This study did not verify any interaction between these factors that could contribute to a limitation in the analysis and interpretation of the data. Considering the limiting effects that some age-related eye diseases pose to patients in their daily activities, future research aiming to analyze the impact of stroke in patients with cataracts, glaucoma and macular degeneration, for example, may contribute to the development of effective therapeutic measures. Following stroke, the importance of ophthalmic evaluation should be considered during routine care of these patients to prevent complications and reduce the degree of disability.

## Conclusions

The data obtained from this study suggest a relationship between stroke and disorders in the detection of spatial frequencies, emphasizing the importance of CSF as a prognostic tool for relevant patients. CSF is associated with the analysis of functional perturbations and the main difficulties found in performing everyday activities.

## Competing interests

The authors declare that they have no competing interests.

## Authors' contributions

NAS conceived of the study and participated in its coordination and helped to draft the manuscript. SMA participated in the design of the study and performed the statistical analysis and drafted the manuscript. All authors read and approved the final manuscript.

## Funding

This paper was funded by National Counsel of Technological and Scientific Development (CNPq), Brazil – Process # 303822/2010-4.

## Pre-publication history

The pre-publication history for this paper can be accessed here:

http://www.biomedcentral.com/1471-2377/12/90/prepub
